# Human Induced Rotation and Reorganization of the Brain of Domestic Dogs

**DOI:** 10.1371/journal.pone.0011946

**Published:** 2010-07-26

**Authors:** Taryn Roberts, Paul McGreevy, Michael Valenzuela

**Affiliations:** 1 Faculty of Veterinary Science, University of Sydney, Sydney, New South Wales, Australia; 2 School of Psychiatry, University of New South Wales, Sydney, New South Wales, Australia; 3 Brain and Ageing Research Program, Faculty of Medicine, University of New South Wales, Sydney, New South Wales, Australia; Universidade Federal do Rio de Janeiro, Brazil

## Abstract

Domestic dogs exhibit an extraordinary degree of morphological diversity. Such breed-to-breed variability applies equally to the canine skull, however little is known about whether this translates to systematic differences in cerebral organization. By looking at the paramedian sagittal magnetic resonance image slice of canine brains across a range of animals with different skull shapes (N = 13), we found that the relative reduction in skull length compared to width (measured by Cephalic Index) was significantly correlated to a progressive ventral pitching of the primary longitudinal brain axis (r = 0.83), as well as with a ventral shift in the position of the olfactory lobe (r = 0.81). Furthermore, these findings were independent of estimated brain size or body weight. Since brachycephaly has arisen from generations of highly selective breeding, this study suggests that the remarkable diversity in domesticated dogs' body shape and size appears to also have led to human-induced adaptations in the organization of the canine brain.

## Introduction

The domestic dog, *Canis familiaris*, exhibits more morphological variation than any other species. Through human selection, breeds have diverged significantly from the form of their closest ancestor, the grey wolf (C*anis lupus*), with the greatest variation evident in the size and shape of the skull [1], which range from 7 to 28 cm in length [2]. Wolves are dolichocephalic (long skulled) but not as extreme as some breeds of *Canis familiaris*, such as greyhounds and Russian wolfhounds (Borzois) [2]. Canine brachycephaly (short-skulledness) is found only in domestic dogs and is related to paedomorphosis in these animals [3]. Puppies of all breeds are born with short snouts, and so the longer skull of dolichocephalic animals emerges during post partum development [4].Other morphological differences in head shape between brachycephalic and dolichocephalic dogs include changes in the craniofacial angle (angle between the basilar axis and hard palate) [5], morphology of the temporomandibular joint [6], and radiographic anatomy of the cribiform plate [7].

Little is known about breed-dependent changes in morphology of the domesticated dog brain. For example, the standard veterinary text, *Miller's Anatomy of the Dog*, notes skull differences between brachy- and dolichocephalic dogs but refers only in passing to the brain [1]. In the 1960s, Seiferle presented a comparison of the brains of brachy- and dolichocephalic dogs, and his diagrams depict brachycephalic brains that are rounded and shortened in the anterior-posterior plane, with a pronounced shift in the position of the olfactory lobe [8].There has since been little attention to the neuromorphological changes in brachycephalic dogs, or what effects these may have on canine behavior or health. At a behavioral level, brachycephaly may be associated with an increased ability to focus and respond to human pointing gestures [9], potentially due to differences in retinal ganglion cell distribution [2]. More generally, reduction in skull length in carnivores correlates with a reduction in olfactory lobe size, hypothetically due to restriction in the development of frontal brain regions [10].

Canine brain research has thus far focused on clinical reports of breed specific disorders, such as pug encephalitis [11], or syringohydromyelia in Cavalier King Charles spaniels [12], or of comparisons between two or three breeds on a given morphological metric, and often these studies have used only one breed to represent a skull type. While the comparison of skull extremes is informative, it would also be of value to investigate whether morphological differences in skull shape across a wide variety of breeds are accompanied by differences in brain organization. Cephalic index (CI) is a simple and useful method of characterizing skull morphology, calculated by dividing skull width by skull length [2], [9], [13], [14], and its use allows an examination of brain organization across the full continuum of dog skull shapes.

In the current study, our aim therefore was to examine the effect of differences in the shape of the canine skull on spatial organization of the brain, focusing on the relationship between the olfactory lobe and supratentorial (above the cerebellum) brain mass, as well as changes of the long axis of the brain. We analyzed paramedian sagittal magnetic resonance image (MRI) slices taken from dogs across a wide range of cephalic indices and developed a number of mathematical measures for capturing these relationships.

## Methods

### Subjects

Eleven recently euthanized dogs of different breeds obtained from a local pound were used in this study. Euthanized dogs from local dog shelters are sometimes used for teaching veterinary anatomy at the University of Sydney as permissible under NSW law, and the university's Animal Ethics Committee confirmed in writing to MV that use of such dogs for the purpose of our study did not require specific committee approval. The investigators had no influence on the fate of these dogs, and conducted no ante-mortem selection or interaction with the individual animals. Deceased animals were MRI scanned within four hours *post mortem* prior to routine cremation. The age of these subjects was not known, and their dominant breed was determined by experienced veterinarians. Two live dogs (both English springer spaniels) were also scanned with owner consent, and were given clearance by the University of Sydney Animal Ethics Committee as part of a larger canine brain ageing study (Ethics approval N00-3-2007-6-4571), resulting in a total sample of N = 13 dogs. None of the dogs in this study were markedly under- or over-weight.

### MR Imaging

Imaging was conducted at the University of Sydney Veterinary Teaching Hospital using a 0.25 Tesla Esaote Vet Grande MRI System (Software release 9.2) with a gradient strength of 20mt/meter and a resonance frequency (RF) strength of 900 watts. All dogs were positioned in sternal recumbency, using RF dual phase array C2 or C4 coils, with the exception of Dog 9 which was scanned in lateral recumbency with a RF linear C1 coil due to large cranial size.

Spin Echo TE Sagittal T1 images were obtained for all 13 dogs (TR 610/TE 18, 3/0.3 mm slice thickness, FOV/RFOV 250×250, 1 NEX and a 250×24 Matrix) with the brain positioned at the isocentre. If the tip of the nose was not visible on the first sagittal scan, dogs were repositioned in the coil with the hard palate at the isocentre and scanned with the same protocol to permit measurement of hard palate angulation.

### Cephalic Measurements and Skull Type

**Figure 1:**
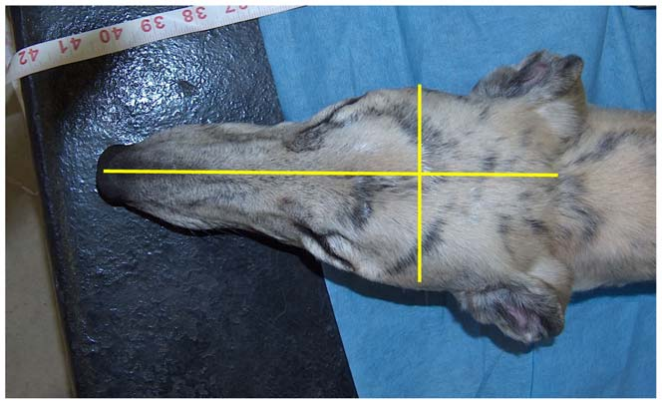
Cephalic Index Measurement. For measurement of the cephalic index, skull width was measured from one zygomatic arch to the other and skull length was measured from the nose to the occipital protuberance. Cephalic index (CI) was calculated as (skull width/skull length) ×100.

Skull length and width were measured on intact dog heads using digital calipers. Skull width was measured at the widest point of the zygomatic arches, and skull length from the external occipital protuberance to the tip of the nose (Figure 1). Cephalic index (CI) was calculated as (skull width/skull length) ×100 [2]. Since the range of cephalic indices defining domestic dog skull types as *dolichocephalic*, *mesocephalic* or *brachycephalic* are not entirely consistent across the literature [1], [15], [16], [17], high- vs low CI grouping was based on median split in our analyzes.

For the purpose of comparison, we were interested in any documented accounts of CI in the wolf (Canis lupus), but could find none. We were, however, able to estimate CI on the basis of two independent sources: 1) www.skullsite.co.uk – an amateur collection of various different animal skulls, including a wolf skull picture and corresponding morphological measurements, and 2) Multi-planar specimen pictures of a gray wolf skull archived by the University of Michigan's Museum of Zoology [18].

### MR Image Analysis

Using Analyze (Biomedical Imaging Resource), each dog's brain was realigned to the hard palate, as per veterinary radiological convention [2]. A paramedian sagittal slice depicting the olfactory lobe at its most distinct was selected and the region of interest of the brain and olfactory lobe were manually traced. Only the supratentorial cerebral hemisphere was traced due to ambiguities in demarcation of the brainstem on sagittal imaging. Planimetric 2D estimates of cerebral size and olfactory volume based on these traced images were calculated using Analyze. Traced images were then saved in Portable Networks Graphic (.png) format and imported into Matlab (MathWorks), for calculation of the centre of mass of the brain (CoM_brain_) and olfactory lobe (CoM_OL_).

**Figure 2:**
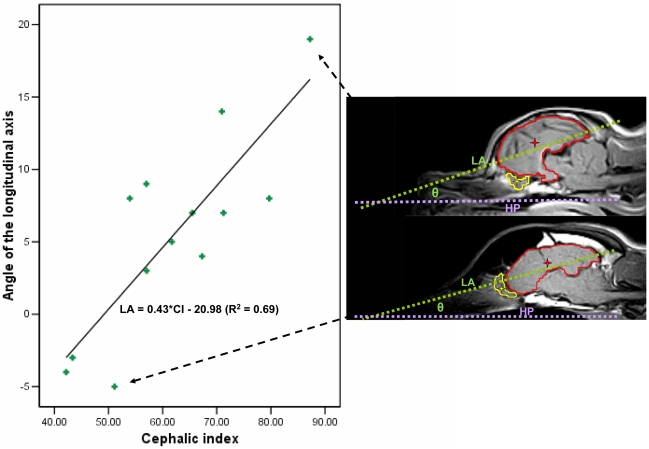
Pitching of the Primary Longitudinal Brain Axis. Cephalic index and longitudinal axis with respect to the hard palate. Individual sagittal scans for dogs at each extreme are shown with the brain outlined in red and centre of mass indicated by a red star. The olfactory bulb has also been outlined in yellow and centre of mass shown in yellow star. HP: Hard Palate reference line. LA: Longitudinal axis. θ  =  Angle of interest.

We then established a longitudinal axis (LA), as the longest possible line drawn from the most rostral point of the frontal lobe to the furthest caudal point of the occipital lobe (Figure 2). The angle of deflection between the hard palate and LA was used to calculate *pitch*, in effect a measure of dorsal-ventral cerebral axis rotation.

**Figure 3:**
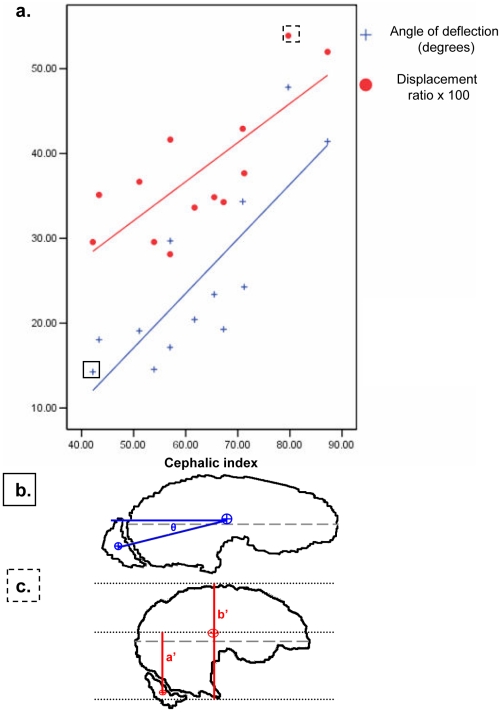
Deviation of the Olfactory Lobe. a) Cephalic index and deviation of the olfactory lobe using two different methods after normalization of cerebral axis to horizontal. Two exemplar dogs highlighted in boxes are illustrated in parts b) and c) below. b) Angle of deflection method: angle in degrees between centre of mass of brain (CoM_brain_) and centre of mass of olfactory lobe (CoM_OL_). c) Displacement method: Ratio of the ventral-dorsal distance from centre of mass of brain and centre of mass of olfactory lobe (a′) to overall brain height (b′).

To analyse the position of the olfactory lobe relative to the cerebral hemisphere, all brains were now realigned to the LA, in effect normalizing for any differences in brain pitch. We measured the angle of deflection of a line drawn between the CoM_OL_ and the CoM_brain_ *relative to the LA axis* (Figure 3b) using Microsoft Picture Manager and the program Universal Desktop Ruler (Version 3.3.3269, AVP Soft, www.AVPSoft.com).

The angle of deflection between the CoM of the brain and that of the olfactory lobe was then calculated. Because variation in brain shape was observed and may have biased deflection measurements, a second method of characterization was also used: a dorsal-ventral linear displacement ratio between CoM_OL_ and the CoM_brain_.

As MRI slices were 3 mm thick, only one suitable slice depicting a distinct olfactory lobe was available for most dogs. The area contained within manual traces of the olfactory bulb was multiplied by the slice thickness to estimate *olfactory bulb volume*. In those two dogs where two slices through the olfactory bulb were present, the average was used. Furthermore, in these two dogs, choice of slice had a negligible effect on calculation of CoM, leading to angle measurement differences of less than 2 degrees.

### Statistical analyses

Analysis was performed using the Statistical Package for the Social Sciences (PASW 18.0 for Windows, SPSS inc, www.spss.com). Pearson correlations were calculated between skull shape, body weight and height, angle of the longitudinal axis and deviation of the olfactory lobe. Because of the small sample size of the brachycephalic and dolichocephalic groups, to test for effects of low or high CI we divided the CI range at the mid-point of CI scores present in this study (CI_mid_ = 64.5) and performed independent sample t-tests to look for differences in angle of the longitudinal axis and deflection of the olfactory lobe between low (CI 42.17–61.70, n = 7) and high (CI 65.48–87.23, n = 6) CI groups.

## Results

### Animals

The type of dogs in this study and their morphological and intracranial measurements are summarized in Table 1. Whilst dogs in the high CI group (i.e., brachycephalic end of spectrum) tended to be smaller and weigh less, none of these comparisons were significantly different.

**Table 1:** Characteristics of dogs in this study by cephalic index group (N = 13).

	High CI (brachycephalic)	Low CI (dolichocephalic)	T-test statistic	p-value
**N**	6	7	–	–
**Male**	50%	43%	–	–
**Breeds**	Akita cross, Mastiff cross, Maltese, Staffordshire bull terrier, Shih tzu cross	Greyhound, English springer spaniel, Australian cattle dog cross, Jack Russell terrier, Pit bull cross	–	–
**Sagittal brain size (mm^2^)**	1475.21±369.4	1722.3±358.6	1.31	0.217
**Olfactory lobe volume (mm^3^)**	255.9±114.3	334.2±110.4	1.26	0.234
**Body weight (kg)**	14.9±9.7	21.3±7.5	1.33	0.211
**Height (cm)**	40.8±13.9	55.3±12.9	1.95	0.78

CI: cephalic index. Mean values ± SD.

### Morphology

Cephalic index (CI) in domestic dogs ranged from 42.2 in a greyhound to 87.2 in a shih tzu cross. In general, body size and weight were closely related to CI in the domestic dogs studied in this sample (see inter-correlations in Table 2). Body weight was therefore used as a covariate in subsequent analyzes.

**Table 2:** Pearson Correlation Data for Skull Shape, Body Size, Body Weight, Angle of the Longitudinal Axis and Deflection of the Olfactory Lobe for N = 13 Dogs.

	Skull Length (mm)	Skull Width (mm)	Body weight (kg)	Body height (cm)	Angle of the Longitudinal axis	Deflection of the olfactory lobe (angle)	Deflection of the olfactory lobe (distance ratio)
**Cephalic Index**	−.839**	−.093	−.661*	−.762**	.828**	.814**	.763**
**Skull Length (mm)**		.591*	.945**	.953**	−.771**	−.927**	−.861**
**Skull Width (mm)**			.708**	.554*	−.196	−.562*	−.550
**Body weight (kg)**				.954**	−.665*	−.842**	−.765**
**Body height (cm)**					−.671*	−.846*	−.796**
**Angle of the Longitudinal axis**						.604*	.493
**Deflection of the olfactory lobe (angle)**							.971**

**: Correlation is significant at the 0.01 level (2-tailed).

*: Correlation is significant at the 0.05 level (2-tailed).

In the two wolf records we could find, CI varied between 50.9 (137 mm/269 mm *100 based on www.skullsite.co.uk measurements) to 51.9 (8.3AU/16AU *100 based on University of Michigan figure).

### Intracranial Volume Estimates

There were no significant differences between dogs in the high versus low CI groups in terms of estimated brain size or olfactory lobe volume (see Table 1).

### Cerebral Axis Rotation

Upon observing the midsagittal images, it was apparent even to the naked eye that dogs with the most brachycephalic skulls had markedly rotated cerebral hemispheres, with the brain pitched ventrally at the anterior pole. This was confirmed quantitatively, as can be seen in Figure 2. There was a significant correlation between pitch rotation and CI (r = 0.828, p<0.001, N = 13). This relationship was not eliminated when either controlling for body weight (partial correlation  = 0.69, p = 0.012, df = 10) or estimated brain size (partial correlation  = 0.72, p = 0.009, df = 10).

This association appeared to be more specific to rostral-caudal skull length (r = −0.771, p<0.002, N = 13), rather than with skull width. For every mm of attenuated skull length relative to width, the canine brain pitched ventrally at the anterior pole by 0.43 degrees. The average angle of the LA Axis of the low CI group (mean: 1.86, SD = 5.84, n = 7) and the high CI group (mean: 9.83, SD = 5.57, n = 6) were significantly different (t = 2.507, 95%CI of mean difference: −14.98 – −0.97, p = 0.029).

### Olfactory-Brain Deflection

Equally striking on the midsagittal images of the dogs scanned in this study was a repositioning of the olfactory lobe relative to the rest of the brain in dogs with reduced skull length. This deflection of the olfactory lobe from the longitudinal axis correlated significantly with CI when measured both in terms of angular deflection (r = 0.814, p = 0.001, N = 13 **Fig 3a,b**) and displacement ratio (r = 0.763, p = 0.002, N = 13, **Figure 3a,c**). The correlation between CI and angular deflection was not eliminated when controlling for either body weight (partial correlation  = 0.64, p = 0.026, df = 10), estimated brain size (partial correlation  = 0.66, p = 0.02, df = 10), or olfactory lobe volume (partial correlation  = 0.73, p = 0.007, df = 10).

Both measures of deviation correlated negatively with skull length (angle r = −0.927, p = 0.000, n = 13; displacement r = −0.861, p = 0.000, n = 13) and the angle of deflection correlated negatively with skull width (angle r = −0.562, p = 0.046, n = 13). The low CI group (mean = 19.0, SD = 5.22, n = 7) and the high CI group (mean = 31.8, SD = 11.31, n = 6) displayed significantly different average olfactory angular deflection values (t = 2.677, 95%CI of mean difference: −23.18 – −2.26, p = 0.022). Individual animals' skull and cerebral morphology measurements are presented in Table 3.

**Table 3:** Individual skull and brain measurements for dogs in this study (n = 13) in the current series.

	Breed	Sex	CI	CI group	Skull length (mm)	Skull width (mm)	Angle of the LA	Deviation of the Olfactory Lobe (Angle)	Deviation of the Olfactory Lobe (Displacement)
1	Greyhound	F	42.17	L	239.5	101	−4	14.26	29.56
2	Greyhound	M	43.36	L	260.6	113	−3	18.05	35.12
3	English springer spaniel	F	51.11	L	229.5	117	−5	19.09	36.67
4	English springer spaniel	F	53.93	L	214.0	115	8	14.54	29.57
5	Australian cattle dog cross	M	57.00	L	216.5	123	9	17.15	28.12
6	Jack Russell terrier	M	57.04	L	163.4	93.2	3	29.7	41.64
7	Pit bull cross	F	61.70	L	216.7	134	5	20.42	33.63
8	Akita cross	M	65.48	H	205.4	135	7	23.39	34.85
9	Mastiff cross	M	67.28	H	222.2	150	4	19.28	34.27
10	Maltese	F	70.94	H	120.1	85.2	14	34.33	42.92
11	Staffordshire bull terrier	F	71.24	H	186.7	133	7	24.27	37.67
12	Shih tzu cross	F	79.69	H	116.7	93	8	47.81	53.89
13	Shih tzu cross	M	87.23	H	113.6	99.1	19	41.42	51.98

Low (L) and High (H) Cephalic index (CI) group based on median split. LA: longitudinal axis.

## Discussion

Our study introduces two new observations about the organization of the brain of the domestic dog. Approximately 69% of the variance in overall pitch of the brain, and 66% of the variance in the relative position of the olfactory lobe, was explained by skull shape as revealed by cephalic index. Increasingly brachycephalic dogs were found a have a more ventrally rotated cerebral axis and a more ventrally shifted olfactory bulb position. Interestingly, these relationships appear to be highly sensitive to CI because rather than appearing beyond a critical threshold, they were found across a wide range of skull shapes and were independent of body weight or brain size.

Canine brachycephaly is purely a human invention. For example, to the best of our knowledge the cephalic index of the wolf (*Canis lupus*) approximates 51 to 52, whilst in our sample of domesticated dogs, ranged from 42 to 87. A complex interplay of breed pressures since canines began human cohabitation about 12,000 years ago [19]– including selection for behavioral, functional, and more recently, aesthetic traits – has led to their amazing physical diversity [9], [20]. Some have speculated as to whether this prepotent physical variation intimates a unique level of plasticity in the canine genome [21]. Added to this, no other animal has enjoyed the level of human affection and companionship as the dog, nor undergone such a systematic and deliberate intervention in its biology through selective breeding.

This diversity is no less prominent than in the wide variation in the shape and size of the canine skull. In this study, this variability was found to extend to the organization of the canine brain. We found a strong correlation between high CI and both cerebral axis rotation (ventrally at the anterior pole) and a ‘ventralization’ of olfactory lobe location. Our analysis suggested this was most strongly associated with skull shortening rather than loss of skull girth in increasingly brachycephalic dogs.

But how could skull shortening affect cerebral organization? Studies of human craniosynostosis [22], [23] and immature head banding [23] suggest that the development of brain shape and size is closely interrelated to the configuration of dura matter as well as the co-developing cranial vault. Changes to any of one of these factors can lead to changes in the others [22]. Differences in canine skull length resulting from artificial human selection pressures may have led to alterations in cerebral development most evident in brachycephalic versus dolicocephalic dogs. Specifically, rostral intracranial volumetric restriction during development of short-skulled dogs may explain the combination of axis rotation and olfactory bulb repositioning. Regodon et al (1993) also noted that reduced skull length in brachycephalic dogs gives rise to a more perpendicular development of the cranium relative to the facial axis [5]. These anatomical adaptations could hence represent a biological solution to a ‘space problem’. The olfactory bulb seems to have migrated to a potential space ventral to the orbital frontal cortex, thereby freeing the anterior pole for normal development of the frontal cortex. Alternatively, animals at the dolichocephalic end of the spectrum may have sufficient ‘spare capacity’ in the cranial vault to permit olfactory bulb development almost directly anterior to the frontal lobe. Either of these possible explanations relies on an evolutionary and developmental preference to preserve frontal lobe volume. Future studies could therefore directly compare frontal lobe morphology in brachy- and dolichocephalic dogs.

Because differences in cranial morphology across dog breeds were closely associated with major neuroanatomical changes, whether these also lead to differences in behavior is a major open question. We cannot yet infer whether the progressive cerebral reorganization found in more brachycephalic dogs have direct functional sequelae. Interestingly, brachycephalic breeds are not typically selected for scent work because of poor olfaction assumed due to crowding of ethmoturbinate bones. Our data suggests a second possible explanation related to anterior-posterior compression of the skull and repositioning of the olfactory lobe relative to the rest of the brain. More broadly, dogs with different skull shapes may behave differently [9], but this is not entirely consistent [24]. Improved behavioral measurement of sensory, motor and cognitive function in domestic dogs is therefore a high priority.

Skull-shape dependent changes in the position of the olfactory bulb also predict a fascinating consequence for the adult rostral migratory stream (RMS). The RMS is a track of neural precursors that originates in the subependymal zone of the lateral ventricles and terminates in the olfactory bulb, contributing to neural turnover in this brain structure in both rodents and humans [25], [26]. Whilst its functional significance remains unresolved, neurogenesis within the RMS is closely connected to olfaction [27], [28]. Given the predictable nature of differences in the location of the canine olfactory lobe based on cranial shape, our findings also predict a rule-based change in the spatial course of the RMS within the brain of domestic dogs. Histological confirmation of this prediction, and any possible behavioral implications for olfaction, are of intense interest for future research.

Finally, a potential limitation on our conclusions is that larger dogs generally tend to have larger brains and manifest a more dolichocephalic cranial morphology, and smaller dogs the opposite. The effects of cranial morphology on brain organization may therefore be confused with those of body and brain size. There are, however, two main reasons why this was unlikely to have been a major confounder in our study. Firstly, there were no significant differences in body weight or estimated brain size between our comparison groups. Secondly, since our study may have been underpowered in this respect, we also took care to control for body weight and brain size in our correlational analyses. So even after accounting for brain size or body weight differences, there was strong evidence for a correlation between CI and both cerebral axis and olfactory lobe position. The effects of skull shape on cerebral axis and olfactory lobe position therefore appear to be independent of body or brain size.

To further disambiguate these competing influence on canine brain organization, future research may also profit by studying those interesting dog ‘outliers’ which break the usual body size-cephalic index norm. These include dolichocephalic breeds with low bodyweight (such as the Italian greyhound), and brachycephalic breeds of high bodyweight (such as the Neapolitan mastiff). Use of high resolution 3D MR imaging as often used in human brain studies would also allow more accurate calculation of whole brain volume, as well as possible changes in lobar organization or grey and white matter distribution.

Overall, our findings suggest that the remarkable variability evident in canine morphology is also apparent in the dog's cerebral organization. We found strong and independent correlations between cephalic index and pitching of the long brain axis, as well as ventral positioning of the olfactory lobe. Further investigation of the inter-relationships between skull shape, brain organization and behavior represent fascinating directions for future canine research.
